# Combining Culture-Dependent and Culture-Independent Methods: New Methodology Insight on the *Vibrio* Community of *Ruditapes philippinarum*

**DOI:** 10.3390/foods10061271

**Published:** 2021-06-03

**Authors:** Angela Zampieri, Massimiliano Babbucci, Lisa Carraro, Massimo Milan, Luca Fasolato, Barbara Cardazzo

**Affiliations:** Department of Comparative Biomedicine and Food Science, University of Padova, Agripolis, Viale dell’Università 16, 35020 Legnaro, Italy; angela.zampieri@phd.unipd.it (A.Z.); massimiliano.babbucci@unipd.it (M.B.); lisa.carraro@unipd.it (L.C.); massimo.milan@unipd.it (M.M.); barbara.cardazzo@unipd.it (B.C.)

**Keywords:** culture-dependent and -independent methods, metabarcoding, shotgun metagenomics, *Ruditapes philippinarum*, microbiota, *Vibrio* spp.

## Abstract

Vibrios represent a natural contaminant of seafood products. *V. alginolyticus*, *V. cholerae*, *V. parahaemolyticus* and *V. vulnificus* are the most hazardous species to human health. Given the worldwide consumption of mollusc products, reliable detection of *Vibrio* species is recommended to prevent human vibriosis. In this study, culture-dependent and -independent methods were compared and integrated to implement knowledge of the Manila clam *Vibrio* community composition. Here, 16S and *rec*A*-pyr*H metabarcoding were applied to compare the microbial communities of homogenate clam samples (culture-independent method) and their culture-derived samples plated on three different media (culture-dependent method). In addition, a subset of plated clam samples was investigated using shotgun metagenomics. Homogenate metabarcoding characterized the most abundant taxa (16S) and *Vibrio* species (*rec*A*-pyr*H). Culture-dependent metabarcoding detected the cultivable taxa, including rare species. Moreover, marine agar medium was found to be a useful substrate for the recovery of several *Vibrio* species, including the main human pathogenic ones. The culture-dependent shotgun metagenomics detected all the main human pathogenic *Vibrio* species and a higher number of vibrios with respect to the *rec*A*-pyr*H metabarcoding. The study revealed that integration of culture-dependent and culture-independent methods might be a valid approach for the characterization of *Vibrio* biodiversity.

## 1. Introduction

Vibrios, which are Gram-negative, rod-shaped bacteria, represent a ubiquitous constituent of marine and brackish ecosystems. Within *Vibrio* biodiversity, some species are associated with aquatic animals, while others have proved to be dangerous to human health. Among the Vibrios that affected marine animals, there are species such as *V. anguillarum* and *V. salmonicida* which are pathogenic to farmed fish, while *V. tapetis* is the main etiological agent of disease for molluscs at all life stages [1]. *V. alginolyticus*, *V. cholerae*, *V. parahaemolyticus* and *V. vulnificus* were found to be the species responsible for the most serious human diseases [2].

Given the significant human health hazard represented by the worldwide distribution of *Vibrio*, reliable methods are required to detect and control their biodiversity distribution, particularly in seafood products. Seafood products, in fact, could represent a vehicle for the foodborne disease vibriosis [3,4]. In addition, it is demonstrated in the literature that the emergence of vibriosis could be related to the increase in global seawater temperature [5]. This phenomenon induces changes in *Vibrio* species distribution and the spread of enteropathogenic species into marine ecosystems.

Over the years, the approaches to characterize *Vibrio* biodiversity, with specific concern regarding human pathogenic species, included culture-dependent and -independent methods. The culture-dependent methods of the Food and Drug Administration’s Bacteriological Analytical Manual (FDA-BAM 2004) and those of ISO/TS 21872-1:2017 are recommended by the health organizations [6].

Different selective media are applied to improve the detection and isolation of the main human pathogenic *Vibrio* species. Thiosulfate-citrate-bile salts-sucrose agar (TCBS) medium represents one of the consolidated selective media adopted to isolate some of the potential pathogenic human *Vibrio* spp. [7]. Moreover, CHROMagar Vibrio (CV) appears to differentiate *V. cholerae*, *V.parahaemolyticus* and *V.vulnificus* by exploiting the different colony colors taken in the chromogenic substrate [8]. Unfortunately, the culture-dependent methods present some limitations. Firstly, bacteria grown on specific media represent only a small fraction of the total community [9]. Specifically, only that part of the community for which the metabolic and physiological requirements can be reproduced in vitro can grow on plates [10]. Consequently, the culturing approach fails to reproduce the entire complex bacterial community present in a natural environment or substrate. In addition, because of the different replication times among culturable bacteria, the grown fraction obtained in plates is distorted. Furthermore, it is difficult to isolate *Vibrio* species present in a viable but non-culturable (VBNC) state [11,12]. Specifically, the VBNC state is a phenotype induced by different stress factors, such as low temperature or excessive UV light exposure, which increase the survival and tolerance of bacteria to harsh environmental conditions. It is a reversible state that could potentially restore bacteria in favorable conditions [13].

Despite the well-known limits of culture-dependent methods, the developed specific media for *Vibrio* spp. allow for focused research on pathogenic targets such as the species *V. cholerae*, *V. parahaemolyticus* and *V. vulnificus*, which represent a potential risk for seafood consumers, avoiding the background of resident microbiota [8,14]. In addition, culture-dependent methods provide the recovery of low-abundant taxa that could be lost to culture-independent methods [15]. Moreover, culture-dependent methods offer the possibility to store, by the alive fraction of the bacterial community, single strains of potential human pathogenic *Vibrio* species in order to perform future genomic analyses [6].

Over the years, several studies have applied PCR-based techniques as culture-independent methods for directly extracting DNA from samples instead of culture in order to obtain more realistic knowledge of the vibrios community composition or of a specific lineage, such as that belonging to human pathogenic *Vibrio* species [16]. For this purpose, in *Vibrio* research, next-generation sequencing (NGS) technology, a PCR-dependent method, has been applied to investigate *Vibrio* biodiversity in environmental and seafood samples. In particular, NGS analysis conducted on 16S rRNA combined with the sequencing of housekeeping genes such as heat shock protein 60 (*hsp60*) and *rec*A-*pyr*H allowed for the identification of *Vibrio* species that naturally contaminate water, oyster, and Manila clam samples [17,18,19]. Moreover, this approach was used to successfully identify potential human pathogenic species such as *V. alginolyticus*, *V. cholerae*, *V. parahaemolyticus* and *V. vulnificus*. PCR-dependent techniques, such as metabarcoding, are a valid tool to investigate complex microbial communities, such as the one related to the food matrix [20]. One of the disadvantages of PCR-dependent techniques is that they do not discriminate alive from dead bacteria [21]. Moreover, amplicon-based methods present an intrinsic bias in the amplification step [22]. Designing inclusive universal primers located in regions more informative than 16S for species/strains’ identification is challenging, and only parts of species/strains are successfully amplified [17,18,19]. The PCR bias can be overcome by using a shotgun metagenomics approach. Shotgun metagenomics has the advantageous capability to sequence all the DNA extracted from a sample, but it requires a huge amount of reads to obtain a reasonable coverage of the microbial genomes as a consequence of the predominance of host DNA [23,24]. There are several commercial kits available to remove host DNA, acting prior to DNA extraction (pre-extraction methods) or after DNA extraction (post-extraction methods), but most of them were developed for liquid samples (saliva, blood, milk) from mammalian hosts [25].

Even if culture-dependent and -independent methods present specific limits, as previously described, a combination of these two approaches could still provide a more comprehensive and accurate overview of bacterial community compositions, as suggested by several studies [26,27]. Following this idea, the objective of this study was to explore the *Vibrio* community composition of *Ruditapes philippinarum* microbiota by combining culture-dependent and -independent methods. Specifically, DNA metabarcoding, developed in a previous study [19], was applied to evaluate and compare the accuracy of isolation and discrimination of *Vibrio* species identified on homogenate clam samples and on their culture-derived samples plated on marine agar (MA), thiosulfate-citrate-bile salts-sucrose agar (TCBS) and CHROMagar Vibrio (CV) media. On a subset of these culture-derived clam samples, shotgun metagenomics was applied in order to enhance the knowledge of the *Vibrio* spp. biodiversity present in Manila clam microbiota.

## 2. Materials and Methods

### 2.1. Sample Collection and Experimental Design

**Figure 1:**
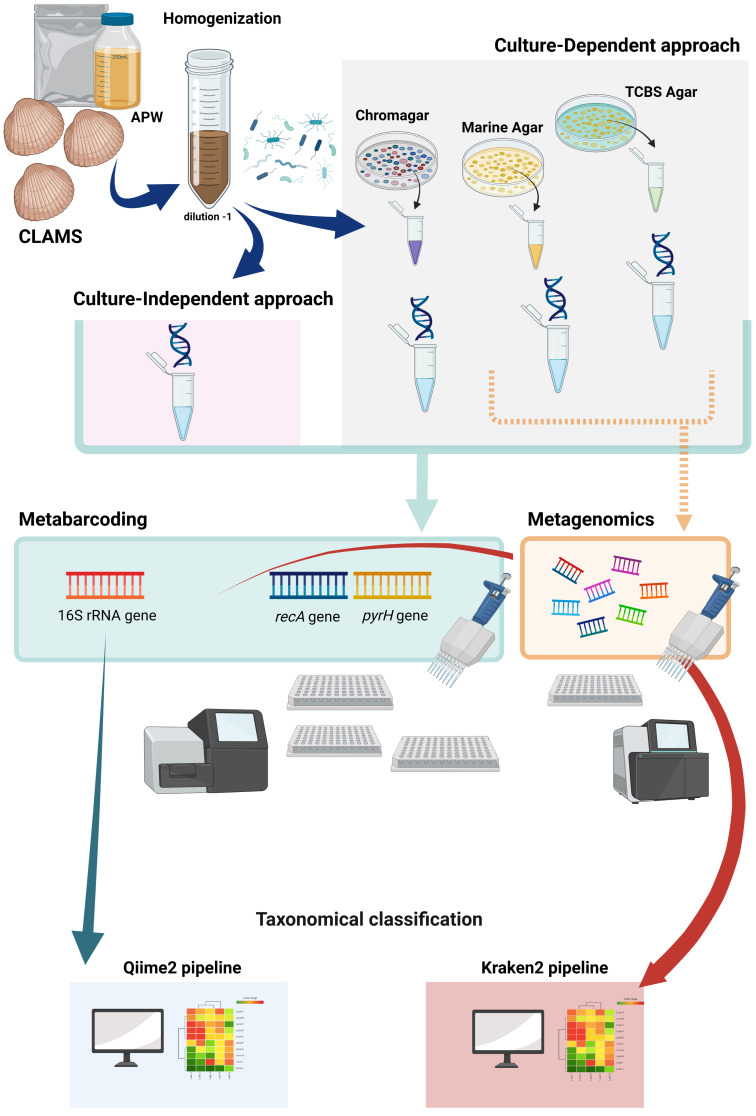
Flow chart of the experimental design. APW: alkaline peptone water; TCBS agar: thiosulfate-citrate-bile salts-sucrose agar.

*Ruditapes philippinarum* individuals of commercial size (weight: 12.9 ± 4.5 g; shell size: 36.2 ± 2.9 g; age: 20 months) were collected from six clam-farming sites along the northeast coast of the Adriatic Sea: (from north to south) Marano (MA), Porto Marghera (PM), Colmata (CO), two sites in Chioggia (CH), Scardovari (SC) and Goro (GO). Sample collection was performed during the summer and the winter seasons. For each site, the batch of clams was analyzed before and after the depuration treatment. The experimental study was designed in order to use a metabarcoding and a shotgun metagenomics approach to analyze and compare the *Vibrio* community composition of isolates from the plate with the first serial dilution of each medium (see Section 2.2), called plated clam samples (culture-dependent method), with the counterparts provided by the clam homogenate samples (culture-independent method). The culture-dependent method adopted three different growth media to define marine bacteria, vibrios and potential human pathogenic *Vibrio* species such as *V. alginolyticus*, *V. cholerae*, *V. parahaemolyticus* and *V. vulnificus*. The investigation on the *Vibrio* community was performed on both clam homogenate and plated clam samples through 16S rRNA and *rec*A-*pyr*H metabarcoding. In addition, the community composition of isolates collected on MA and TCBS plates from 16 samples was investigated using shotgun metagenomics (Figure 1, created with Biorender.com). In total, 54 homogenate clam samples were obtained, of which 26 were collected in the summer season and 28 in the winter season (Table S1, Supplementary Materials).

### 2.2. Microbiological Analysis

Clams were processed as described in a previous study of Zampieri et al. [19], with the addition of a microbiological analysis performed using a selective medium coupled with the temperature of incubation required for the detection of the main human pathogenic *Vibrio* species. Briefly, clams were scrubbed under running potable water and the shell cleaned with ethanol 100%. Subsequently, clams were weighted, measured and shucked to collect 25 g of flesh and intervalvular liquid clam tissues into a sterile stomacher bag. For the homogenization, 225 mL of alkaline peptone water (APW, 2% NaCl, 1% peptone, pH 8.5; Microbiol, Macchiareddu, CA, USA) was added and then tenfold serial dilution was performed. Subsequently, for samples of the summer season, 100 µL of each serial dilution was plated on marine agar (MA) (Condalab, Cagliari, CA, USA) and thiosulfate-citrate-bile salts-sucrose agar (TCBS) (Biolife, Monza, MI, USA) media. For samples collected during the winter season, in addition to TCBS and MA media, 100 µL of each serial dilution was plated on CHROMagar Vibrio media (CV) (CHROMagar Microbiology, Paris, France) for the isolation of *V. cholerae*, *V. parahaemolyticus* and *V. vulnificus*. The plates of TCBS and CV media were incubated at 22 and 37 °C for 24–48 h [28]. The MA plates were incubated at 22 °C for 24 h. After incubation was completed, the total bacterial communities grown on MA, CV and TCBS media (dilution -1) were gathered by scrubbing and washing the surface of the plates with 2 mL of phosphate-buffered saline (PBS). Then, suspended cells were centrifuged at 10,000 rpm for 1 min (Eppendorf centrifuge 5424) and the pellets were stored at −80 °C until performing the DNA extraction. For determination of the homogenates’ microbial community, from each homogenate, an aliquot of 2 mL was collected and then centrifuged at 10,000 rpm for 1 min to obtain a pellet (Eppendorf centrifuge 5424). Then, the pellets were stored at −80 °C until performing the DNA extraction for culture-independent analysis.

### 2.3. DNA Extraction and Libraries’ Preparation for Metabarcoding

DNA samples of the microbial communities collected from the MA, TCBS and CV media were extracted using the Invisorb^®^ Spin Tissue Mini Kit (Invitek molecular, GMBH, Berlin, Germany) following the manufacturer’s instructions. For the microbial community of homogenates, DNA was extracted using a DNeasy^®^ PowerSoil^®^ Kit (Qiagen, Hilden, Germany) following the manufacturer’s instructions. As described in a previous study [19], a mock community DNA sample was included into the sequencing libraries analysis as an internal control. Subsequently, each DNA sample was diluted for Illumina library preparation, performed on 16S rRNA, *rec*A and *pyr*H genes. Specifically, the DNA of homogenates was diluted to 1:5, while DNA from each plated clam sample was diluted to reach 2 ng/µL. Illumina libraries were prepared using a two-step approach as described in a previous study [19]. PCR composition and thermal profiling was conducted as reported in [29], using, in the first PCR step, the specific temperature of annealing for the *rec*A and *pyr*H genes as indicated by Zampieri et al. [19]. The result of each PCR step was verified on agarose gel 1.8% and purified using the SPRIselect reagent Kit (Beckman Coulter Genomics) following the manufacturer’s instructions. Quantification of the purified samples was conducted using a Qubit ^TM^ dsDNA BR Assay Kit (Invitrogen, Life Technologies, Monza, Italy)) as an end-point fluorometric detection. The equimolar pool of the final libraries was checked for the quality using an Agilent 2100 Bioanalyzer (Agilent Technologies, Palo Alto, CA, USA) and for the quantification using a Qubit^®^ Assay Kit BR. The 16S rRNA, *rec*A and *pyr*H libraries were sequenced at the UC Davis Genome Center (California) using the MiSeq System, Illumina (300 bp forward and reverse). The raw sequence data were deposited in the NCBI database with the following BioProject ID: PRJNA726587.

### 2.4. Shotgun Metagenomics Libraries Preparation

For a subset of 16 plated clam samples, DNA quantity control was performed using a Qubit dsDNA HS Assay (Invitrogen, Life Technologies, Monza, Italy). The library construction was assessed using a Nextera XT DNA Sample Preparation Kit (Illumina, Inc., San Diego, CA, USA) with IDT for Illumina Nextera DNA UD Indexes. Then, a 2100 Bioanalyzer (High Sensitivity DNA Assay, Agilent Technologies) was used to verify the quality of the libraries. Finally, the libraries were run on an Illumina Novaseq Sp500 PE250 (Illumina, Inc., San Diego, CA, USA). The raw sequence data were deposited in the NCBI database with the following BioProject ID: PRJNA726531.

### 2.5. Bioinformatic and Statistical Analyses for Metabarcoding

Firstly, the quality of the raw reads of homogenates and plated clam samples was checked and visualized using FastQC software (version 0.11.9). Subsequently, the bioinformatics analysis procedure followed that described in a previous study [19]. In brief, 16S rRNA raw sequence data were trimmed and merged with DADA2, and a full analysis was conducted using the QIIME2 platform (https://qiime2.org/, QIIME2 version accessed on 10 February 2021). To assign taxonomy, a Naïve Bayes classifier was employed using the SILVA138 release data. After trimming and filtering using Trim Galore v. 0.6.6 (https://www.bioinformatics.babraham.ac.uk/projects/trim_galore/, accessed on 10 April 2020), the *rec*A and *pyr*H sequences were imported to Kraken 2 software (https://ccb.jhu.edu/software/kraken2/, accessed on 10 February 2020) to perform a full data sequencing analysis against the MiniKraken2_v1_8 GB database [30]. Then, Bracken software was used to carry out a Bayesian inference of abundance of the species detected during data sequencing (https://ccb.jhu.edu/software/bracken/, accessed on 10 February 2020). This software used the taxonomy labels assigned by Kraken 2 to estimate the number of reads deriving from each species present in a sample [31]. As conducted in a previous study [19], a confidence cut-off set at 0.1 was applied in order to define species attribution inside the bacterial communities of homogenates and plated clam samples. In addition, a filter was applied to set the minimum number of reads (10) reliable for *Vibrio* species detection. The homogenate and plated clam samples produced a total of 45,000,000 raw reads and 50,000,000 raw reads for 16S Illumina Miseq and *rec*A-*pyr*H libraries sequencing, respectively. Firstly, a rarefaction curve was drawn to assess the representation of microbial communities defined by 16S rRNA gene. Subsequently, an exploratory analysis of the output files of QIIME2 and Bracken software was carried out using the CALYPSO platform [32]. The microbial alpha diversity of 16S rRNA data sequencing was studied using an ANOVA (*p*-value < 0.05) carried out at the feature level (ASV) according to the Richness Index. Then, the beta microbial diversity was described using a PCoA representation. The statistic test PERMANOVA was used to evaluate the different community compositions according to 16S rRNA (ADONIS +, according to the Bray–Curtis index) and *rec*A-*pyr*H data sequencing (PERMANOVA Pairwise comparisons). As reported in a previous study, *rec*A-*pyr*H merged results were elaborated using a qualitative approach (presence/absence of species) [19]. The number of shared taxa and bacterial species, identified by 16S rRNA and *rec*A and *pyr*H genes, respectively, among homogenate and plated clam samples was visualized through Venn diagrams. Finally, the agreement among the detection of each *Vibrio* species using homogenate (*rec*A-*py*rH culture-independent method) and clam samples plated and incubated at 22 °C on MA and TCBS media (*rec*A-*pyr*H culture-dependent method) was evaluated using kappa statistical measures [33]. Specifically, we used Fleiss’ kappa, for which values were summarized as poor agreement (k < 0), slight agreement (0.0 < k < 0.20), fair agreement (0.21 < k < 0.40), moderate agreement (0.41 < k < 0.60), substantial agreement (0.61 < k < 0.80) and almost perfect agreement (0.81 < k < 1).

### 2.6. Bioinformatic and Explorative Analyses for Shotgun Metagenomics

The shotgun metagenomics libraries yielded a total of 120,000,000 raw reads (forward and reverse) for all samples. The shotgun metagenomics data elaboration started with the application of metaWRAP, a specific pipeline for metagenomic analysis [34], on the de-multiplexed reads. Reads’ trimming and human contamination removal were performed using the metaWRAP Read_qc module (including Trim Galore, Cutadapt and BMTagger tools with default parameters). Taxonomic profiling was performed on the trimmed reads with the metaWRAP Kraken module for metagenomics (cut-off set at 0.1, against MiniKraken2_v1_8 GB database). Bracken (Bayesian Reestimation of Abundance with Kraken) was used to compute the abundance of bacterial species using the taxonomy labels assigned by Kraken. The bracken report files were converted into a biom file by using the Kraken-biom tool. Subsequently, the microbial community composition was investigated with a qualitative assessment based on the presence/absence of species identified. In detail, a heat map was defined using the output Bracken files of the 16 plated clam samples investigated through shotgun metagenomics and the corresponding output Bracken files of the homogenate and plated clam samples investigated through metabarcoding. Specifically, the heat map, based on a matrix file of the presence/absence of detected species, was realized by using ComplexHeatmap package in R (version 4.0.3). As described in a previous study conducted by Zampieri et al. [19], for the creation of the matrix of presence/absence, a reliable filter was used, with a minimum of ten reads. Then, Venn diagrams were generated to visualize the number of shared species among the clam samples plated on MA 22 °C and TCBS 22 °C media according to metabarcoding and shotgun metagenomics.

## 3. Results

### 3.1. 16S Metabarcoding-Based Microbial Communities of Homogenate and Culture-Derived Clam Samples

**Figure 2:**
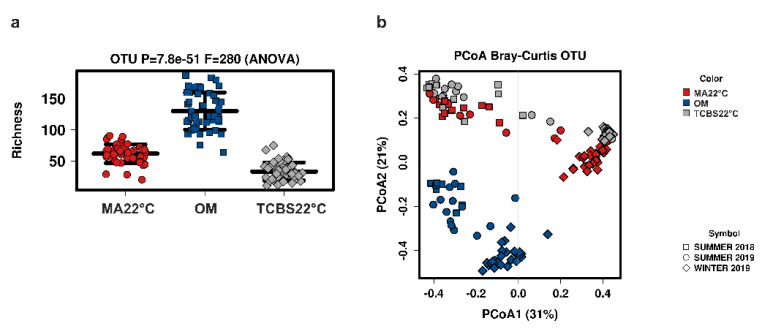
16S amplicon-based microbial diversity of clam samples. (**a**) 16S amplicon-based alpha diversity tested with ANOVA according to Richness Index. (**b**) 16S amplicon-based microbial principal coordinate analysis (PCoA) representation of homogenate and plated clam samples according to the Bray–Curtis Index. In PCoA representation, the season factor corresponding to the collection time of clam samples is also reported. OM: Homogenate clam samples; MA 22 °C: clam samples plated on marine agar and incubated at 22 °C; TCBS 22 °C: clam samples plated on thiosulfate-citrate-bile salts-sucrose agar and incubated at 22 °C.

The microbiota composition of homogenate and plated clam samples was investigated through 16S rRNA amplicon sequencing in order to evaluate the recovery capability of each culture media. The rarefaction curve of filtered reads showed a good representation of the microbial communities (Figure S1, Supplementary Materials). Furthermore, the alpha diversity analysis revealed a higher level of taxa in the homogenate samples with respect to the clam samples plated and incubated at 22 °C on MA and TCBS media. The clam samples plated on TCBS 22 °C media showed a lower level of taxa with respect to the homogenate and clam samples plated on MA 22 °C media (Figure 2a). Then, the variation in the bacterial communities (beta diversity) was explored among the homogenate and the plated clam samples. Communities from CV 22 °C and TCBS 37 °C were not included in this analysis because these media and conditions of incubation allowed the growth of a limited number of taxa. The multivariate analysis demonstrated a different community composition of homogenate samples compared to the plated clam samples (*p*-value < 0.001). Moreover, a different microbial composition was also observed depending on the season, where summer clam samples’ results were different from those of the winter clam samples (*p*-value < 0.001). The variation in the bacterial communities was then visualized with a PCoA representation, according to the Bray–Curtis index. The PCoA representation reported in Figure 2b shows a clear clustering of homogenate samples separated from the clam samples plated on MA 22 °C and TCBS 22 °C media. The communities from the two media instead showed a partial overlapping of samples. In addition, the effect of the season of sample collection clearly separated summer clam samples from the winter clam samples.

**Figure 3:**
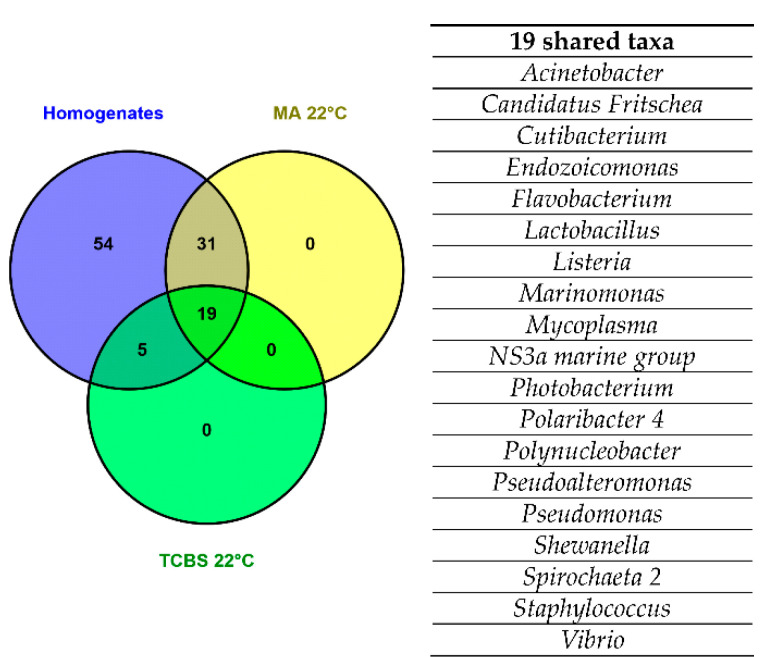
Venn diagram showing the number of taxa, identified by 16S metabarcoding at the genus level, shared among homogenate samples and clam samples plated on MA 22 °C and TCBS 22 °C media. The table report the detail of the nineteen taxa shared among homogenate and plated clam samples. MA 22 °C: clam samples plated on marine agar and incubated at 22 °C; TCBS 22 °C: clam samples plated on Thiosulfate-citrate-bile salts-sucrose agar and incubated at 22 °C.

Subsequently, the number of taxa shared among the homogenate and the plated clam samples was described through a Venn diagram, according to a matrix of the presence/absence of taxa (Figure 3). As previously demonstrated in the alpha diversity analysis, the homogenate samples showed a higher taxa biodiversity, evaluated at the genus level, with respect to the clam samples plated on MA 22 °C and TCBS 22 °C media. In total, 19 taxa were shared among the homogenate clam samples and the clam samples plated on MA 22 °C and TCBS 22 °C media. Specifically, the taxa found in the homogenate samples comprised all the taxa in the clam samples plated on TCBS 22 °C and MA 22 °C media (Figure 3).

### 3.2. recA-pyrH Metabarcoding on Homogenate and Plated Clam Samples

**Figure 4:**
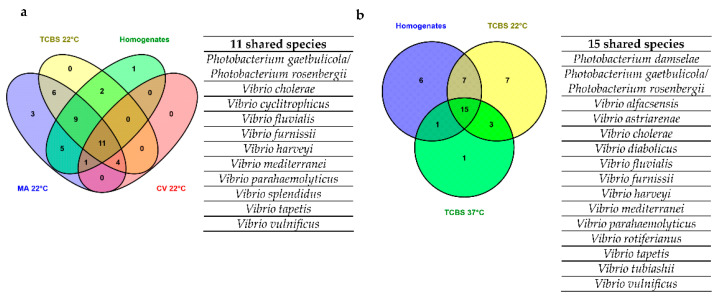
Venn diagram showing the number of *Vibrio* and Vibrionaceae species, identified by *rec*A-*pyr*H metabarcoding, shared among homogenate and clam samples plated on MA 22 °C, TCBS 22 °C and CV 22 °C media (**a**) and among homogenate and clam samples plated on TCBS 22 °C and TCBS 37 °C media (**b**). The two tables report the details of the number and names of shared species among homogenate and plated clam samples. MA 22 °C: clam samples plated on marine agar and incubated at 22 °C; TCBS 22 °C: clam samples plated on thiosulfate-citrate-bile salts-sucrose agar and incubated at 22 °C; CV 22 °C: clam samples plated on CHROMagar *Vibrio* media and incubated at 22 °C.

The 16S metabarcoding provided a description of the complete bacterial community, while *rec*A and *pyr*H sequencing were performed to investigate the *Vibrio* and Vibrionaceae species biodiversity highlighted by the culture-dependent and -independent methods. Firstly, the diversity of microbial species composition (beta diversity) was investigated among homogenate and plated clam samples. Pairwise comparisons, according to PERMANOVA analysis, showed statistical differences in the *Vibrio* community composition between homogenate samples and plated clam samples on MA 22 °C, TCBS 22 °C, TCBS 37 °C and CV 22 °C media (*p*-value < 0.001). In addition, PERMANOVA analysis revealed a different community composition of homogenate samples (culture-independent method) in comparison with metabarcoding on culture media when also considering the season stratification (*p*-value 0.0001). Subsequently, the variation in the *Vibrio* community between samples was visualized using a PCoA representation according to the Jaccard index (Figure S2, Supplementary Materials). Specifically, the PCoA showed a higher dispersion of homogenate and clam samples plated on MA22 °C and TCBS 22 °C media. Clam samples plated on CV 22 °C and TCBS 37 °C media were more clustered with respect to the other clam samples. Moreover, the PCoA representation showed a clear separation of samples collected in winter with respect to the ones collected in the summer season (Figure S2, Supplementary Materials). Then, Venn diagrams were generated to represent the number of *Vibrio* and Vibrionaceae species shared among the homogenate and plated clam samples. Firstly, the number of species shared among homogenate samples and clam samples plated and incubated at 22 °C was studied (Figure 4a). Subsequently, the effect of the two temperatures of incubation (22 and 37 °C) on the number of species shared among homogenate and clam samples plated on TCBS medium was investigated (Figure 4b). In total, 11 taxa including *Photobacterium* and *Vibrio* species were shared among the homogenates and the clam samples plated on MA, TCBS and CV at 22 °C (Figure 4a). Among the *Vibrio* species, the main potential human pathogenic *Vibrio* species, such as *V. cholerae*, *V. parahaemolyticus* and *V. vulnificus*, were detected. All the culture media at 22 °C evidenced taxa that were not identified in the homogenate samples, and MA showed a higher number of *Vibrio* species with respect to the homogenate samples and to the other plated samples. According to the comparison of the homogenates with TCBS samples at the two temperatures of incubation, the homogenates shared 15 taxa, including 13 *Vibrio* species and 2 Vibrionaceae, with plated samples (Figure 4b). Again, the potential human pathogenic *Vibrio* species, such as *V. cholerae*, *V. parahaemolyticus* and *V. vulnificus*, were included among the shared species between homogenates and clam samples plated on TCBS medium. The clam samples plated on TCBS 22 °C showed a higher number of *Vibrio* species with respect to the clam samples plated on TCBS medium and incubated at 37 °C (Figure 4b).

Then, the kappa statistical measures were evaluated among homogenate and clam samples incubated at 22 °C and plated in MA and TCBS media. The kappa statistical measures revealed a significant agreement (*p*-value < 0.05) in the detection of thirteen *Vibrio* species and three Vibrionaceae species (Table S2, Supplementary Material). Specifically, eight *Vibrio* species reported a moderate agreement, and two *Vibrio* species reported a significant statistical agreement in terms of detection among the three approaches. Regarding human pathogenic *Vibrio* species, the detection of *V. parahaemolyticus* and *V. vulnificus* showed a significant moderate agreement among homogenate and plated clam samples. *V. cholerae* and *V. alginolyticus*, on the other hand, showed a slight and not significant agreement among the culture-independent methods and metabarcoding on MA and TCBS, respectively. In addition, among the potential *Vibrio* species pathogenic to molluscs, species such as *V. tapetis* and *V. splendidus* showed a statistically fair agreement (Table S2, Supplementary Materials). Moreover, considering culturable and not culturable approaches, clam samples accounted for a noticeable percentage of human pathogenic *Vibrio* species. *V. alginolyticus* was detected in 16.7%, *V. cholerae* in 66.7%, *V. parahaemolyticus* in 56.3% and *V. vulnificus* in 54.2% of the samples.

### 3.3. Shotgun Metagenomics of Plated Clam Samples and Comparison of Metagenomics and recA-pyrH Metabarcoding Community

Shotgun metagenomics was applied to 16 plated clam samples, including 8 from MA 22 °C and 8 from TCBS 22 °C, in order to obtain more accurate knowledge of the *Vibrio* species community composition. Three clam samples (3A, 7A, 8A) plated on MA medium produced low-quality raw reads during the shotgun metagenomics sequencing and, consequently, were not included in the analysis (Tables S1 and S3, Supplementary Materials). Then, the shotgun metagenomics data were compared to the results obtained by *rec*A-*pyr*H metabarcoding on the same plated clam samples.

**Figure 5:**
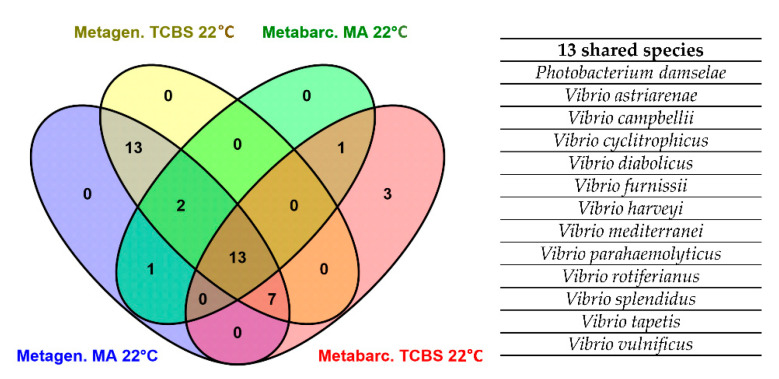
Venn diagram showing the number of *Vibrio* species found in clam samples plated on MA and TCBS media according to the *rec*A-*pyr*H metabarcoding and shotgun metagenomics results. The table reports the details of the thirteen species commonly identified by metabarcoding and shotgun metagenomics. MA 22 °C: clam samples plated on marine agar and incubated at 22 °C; TCBS 22 °C: clam samples plated on thiosulfate-citrate-bile salts-sucrose agar and incubated at 22 °C; Metagen.: shotgun metagenomics; Metabarc.: metabarcoding.

The number of species commonly identified by shotgun metagenomics and *rec*A-*pyr*H metabarcoding was visualized using a Venn diagram. In total, the Venn diagram reported 12 *Vibrio* and 1 Vibrionaceae species commonly identified by metagenomics and metabarcoding in the two media, MA and TCBS (Figure 5). The potential human pathogenic *Vibrio* species, such as *V. parahaemolyticus* and *V. vulnificus*, were among the 13 shared species. In addition, metabarcoding conducted on the eight clam samples plated on TCBS medium identified two *Vibrio* (*V. ponticus*, *V. Scap.*24) and one Vibrionaceae (*P. gaetbulicola/P. rosenbergii*) species not detected by shotgun metagenomics.

**Figure 6:**
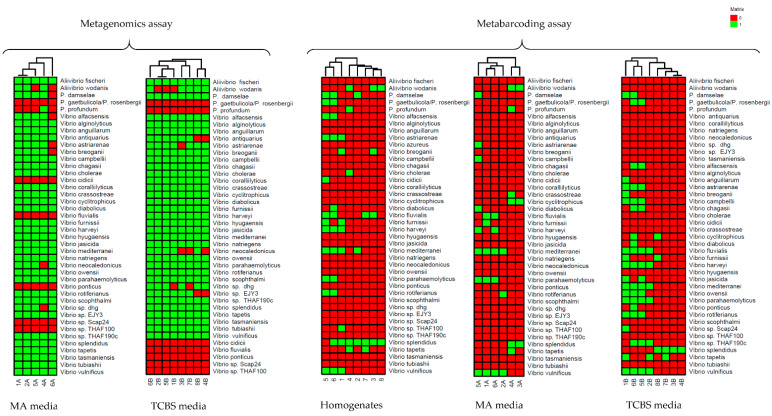
Heat map showing the presence (green) and the absence (red) of Vibrios found in clam samples (homogenates and clam samples plated on MA and TCBS media) according to shotgun metagenomics and *rec*A-*pyr*H metabarcoding. The letter “A” is assigned to samples plated on marine agar (MA) medium; the letter “B” is assigned to samples plated on thiosulfate-citrate-bile salts-sucrose agar (TCBS) medium. A number code (from 1 to 8) is assigned to each homogenate sample. P.: Photobacterium.

From each specific medium, several species were identified by both shotgun metagenomics and metabarcoding. Specifically, in MA and TCBS media, shotgun metagenomics and metabarcoding commonly identified 13 *Vibrio* and 3 Vibrionaceae species (Figure S3, Supplementary Materials) and 19 *Vibrio* and 1 Vibrionaceae species, respectively (Figure S4, Supplementary Materials). Subsequently, these shotgun metagenomics and metabarcoding data were compared to the results obtained by *rec*A-*pyr*H metabarcoding of the homogenates of the same 16 samples. The comparison was visualized using a heat map representation, as reported in Figure 6. Specifically, shotgun metagenomics identified, in all samples, the main potential human pathogenic *Vibrio* species such as *V. alginolyticus*, *V. cholerae*, *V. parahaemolyticus* and *V. vulnificus*. On the contrary, metabarcoding did not find *V. alginolyticus* in the homogenate and plated clam samples. *V. cholerae* was identified only in one homogenate sample (4). Moreover, *V. parahaemolyticus* and *V. vulnificus* were identified by metabarcoding, but not in all of the homogenate and plated clam samples (Figure 6).

Heat map representation was also used to describe the additional species not belonging to the Vibrionaceae family that were detected by metabarcoding and shotgun metagenomics. In particular, shotgun metagenomics identified 26 and 25 marine bacteria species not belonging to the Vibrionaceae family in MA and TCBS media, respectively (Figure S5). Specifically, *rec*A-*pyr*H metabarcoding detected *Litorilituus sediminis* and *Haemophilus parainfluenzae* only in two homogenate samples and *Shewanella sp. Scap07* in one clam sample plated in TCBS medium (7B). These species were not found by shotgun metagenomics (Figure S5, Supplementary Materials).

## 4. Discussion

Seafood products represent reservoirs and vectors for several *Vibrio* species, including the main human pathogenic *Vibrio* species such as *V. alginolyticus*, *V. cholerae*, *V. parahaemolyticus* and *V. vulnificus* [1,35,36,37]. Consequently, an up-to-date knowledge of the *Vibrio* species biodiversity present in seafood products requires accurate methods of detection and identification of these marine bacteria. Over the years, in order to prevent the spread of human vibriosis, the characterization of *Vibrio* species associated with seafood products has been carried out using culture-dependent and/or -independent methods [6]. A recent research field involved in the characterization of microbial communities reported the advantage of the combined use of culture-dependent methods with culture-independent ones. Specifically, these studies highlighted the complementarity of these two methods when applied for the characterization of microbial biodiversity [15,38]. Following this promising coupled approach, in this study, culture-dependent and -independent methods were used in parallel to increase the knowledge on Manila clam *Vibrio* community compositions, with specific concern regarding the *Vibrio* species that are hazardous to human health.

First, the variation in bacterial community composition was evaluated, according to 16S rRNA data sequencing, in homogenate samples (culture-independent method) and clam samples plated on MA and TCBS media (culture-dependent method). In this first evaluation, the community compositions of clam samples plated on TCBS and incubated at 37 °C (TCBS 37 °C) and clam samples plated on CV and incubated at 22 °C (CV 22 °C) were not considered. These media and temperatures of incubation showed specificity and selectivity for the detection of the main human pathogenic *Vibrio* species such as *Vibrio parahaemolyticus* [14,39] and, consequently, did not offer a general overview of the bacterial community composition.

In agreement with Manuel Anguita-Maeso et al. [26], our results suggested that microbial composition is strongly dependent on the use of culture-dependent and -independent methods. The selection performed using the culture-dependent method was corroborated by the higher biodiversity of taxa found in the homogenate samples with respect to the plated clam ones (Figure 2a and Figure 3). This higher biodiversity could be explained by the direct extraction of DNA from the clam homogenate samples, which contributed to the detection of those marine taxa not recovered in the culture media as they were either not alive (or in a dormant VBNC state) or not able to grow in the culture media [40]. Moreover, the different microbial compositions of the homogenate and plated clam samples could derive from the effect of the different selections of cultural media, MA and TCBS, on the taxa present in clam samples (Figure 2b). The non-selective medium MA is commonly applied for the recovery of a wide range of marine bacteria [41]. TCBS medium, instead, is primarily used for the detection of human pathogenic *Vibrio* species such as *V. cholerae* and *V. parahaemolyticus*. Besides this application, TCBS medium was also useful for the recovery of several environmental vibrios [42]. All microbial communities, defined by both culture-dependent and -independent methods, could be well differentiated by season (Figure 2b). This result is in line with several studies in which the seasonal shifts of microbial communities are reported in freshwater and seawater ecosystems [43,44].

The investigation on clam-associated microbiota performed with 16S metabarcoding was completed using *Vibrio*-specific metabarcoding (based on *rec*A-*pyr*H genes) for the characterization of the *Vibrio* community, paying specific attention to the potential human pathogenic *Vibrio* species. Clam samples plated on CV (CV 22 °C) and TCBS media (TCBS 37 °C) were also included in the analysis of *Vibrio* community composition, incubated at 22 and 37 °C, respectively. As expected, PERMANOVA analysis revealed a different *Vibrio* biodiversity community between homogenate and plated clam samples. The PCoA representation of homogenate and plated clam samples, in fact, presented a higher dispersion with the clusters of clam samples plated on TCBS 37 °C and CV 22 °C media (Figure S2). Clam samples plated on TCBS 37 °C and CV 22 °C media, indeed, showed a lower number of *Vibrio* and Vibrionaceae species with respect to the other clam samples plated on TCBS (TCBS 22 °C) and MA (MA 22 °C) media incubated at 22 °C (Figure 4a,b). This result is in accordance with the fact that culture conditions, represented by TCBS and CV media composition coupled with a specific temperature of incubation, are commonly applied to carry out investigations on restricted *Vibrio* biodiversity, represented mostly by the potential human pathogenic *Vibrio* species. The stringent selection performed using TCBS medium incubated at 37 °C represents a step in the ISO/TS 21872-1:2017 standard procedure for the detection, enumeration and isolation of enteropathogenic *V. parahaemolyticus*, *V. cholerae* and *V. vulnificus*. Similarly, CV medium has been applied in several studies to characterize the main human pathogenic species, such as *V. cholerae*, *V. parahaemolyticus* and *V. vulnificus*, by exploiting the chromogenic substrate offered by this medium [8,39]. Curiously, clam samples plated on MA 22 °C medium resulted in a higher number of *Vibrio* species identified compared to the other plated and homogenate samples (Figure 4a). This suggested that MA medium, despite not being inclusive for the general clam microbiota, is a valid substrate for the detection of several environmental *Vibrio* species, including potential human pathogenic species such as *V. alginolyticus*, *V. cholerae*, *V. parahaemolyticus* and *V. vulnificus*. Moreover, the MA medium allowed for characterizing rare species, such as *V. casei*, *V. hyugaensis* and *V. rumoieinsis*, which were not found in the other plated clam and homogenate samples. Consequently, despite the common application of MA medium as a non-selective medium for culturing several marine bacteria, it could also be considered adequate for application, coupled with *rec*A*-pyr*H metabarcoding, for the target isolation of *Vibrio* species. In a recent study, MA medium was applied by the authors as a positive control medium, which showed a comparable growth of environmental vibrios isolates to the one obtained from the modified TCBS medium used in the study [45]. This study demonstrated the advantage of the combined use of culture-dependent and culture-independent methods for the detection of human pathogenic *Vibrio* species (Table S1, Supplementary Materials). Specifically, despite the comparable reliability in the detection of *V. parahaemolyticus* and *V. vulnificus* (significant moderate k agreement), the combination of culture-dependent and -independent methods was necessary for the detection of *V. alginolyticus* and *V. cholerae* (slightly not significant k agreement) (Table S2, Supplementary Materials). Unlike a previous study [19], *V. alginolyticus* was not found in homogenate samples. However, this potential human pathogenic *Vibrio* species was detected by the culture approach in the clam samples plated on TCBS 22 °C, TCBS 37 °C, CV 22 °C and MA 22 °C media. This result could be explained by the low concentration of *V. alginolyticus* on homogenate samples, which, covered by the other outgrown *Vibrio* species, was not found by the *rec*A-*pyr*H metabarcoding. In addition, given the halophilic nature of *V. alginolyticus* species [46], it is possible to assume that the plating step performed on TCBS, CV and MA media, satisfying the salt requirement of this species, promoted the growth and subsequent molecular detection. This hypothesis can be corroborated by the study of Tagliavia et al. [46], in which the authors demonstrated that increasing the salt concentration of a medium used for *Vibrio* spp. detection allows for a more accurate estimation of the actual presence of *Vibrio* species in dilution plate counts.

Moreover, TCBS medium coupled with two different temperatures of incubation (22 °C and 37 °C) was also useful for the detection of several environmental *Vibrio* species not identified in the homogenate samples. Again, the *Vibrio* species not identified in the homogenate samples were probably present in these samples, but at such a low level that they were unable to compete with the other more abundant species, and consequently, they were not detected (Figure 4b). Moreover, only in clam samples plated on TBCS 37 °C medium was *V. mimicus* found. These culturing conditions are required to isolate this species, closely related to *V. cholerae*, which represents a potential risk for marine animals and humans [47]. Moreover, as previously described, the *Vibrio* community composition among homogenate and plated clam samples showed microbial changes depending on the season of sample collection. In line with this result, several studies reported different *Vibrio* biodiversity in seafood products depending on the time of samples’ collection [48,49]. In addition, the kappa statistical measures showed the complementarity of culture-dependent methods coupled with the independent ones in the detection of the juvenile/adult molluscan pathogens *V. tapetis* and *V. splendidus* [50]. Specifically, the homogenate samples and the clam samples plated on MA 22 °C and TCBS 22 °C showed a significant, fair agreement for the detection of these two *Vibrio* species. For these reasons, the application of culture-dependent methods in combination with culture-independent ones is strongly recommended to detect these species. The presence of *V. tapetis* and *V. splendidus* in *Ruditapes philippinarum* microbiota is in accordance with a previous study by Zampieri et al. [19].

In order to obtain a more complete picture of *Vibrio* species biodiversity, in this study, shotgun metagenomics was also applied on 16 MA- and TCBS-plated clam samples. The metagenomics investigation was not applied on homogenate samples because host DNA tends to overwhelm bacterial DNA on sequencing results [51].

Subsequently, the *Vibrio* communities identified by shotgun metagenomics were compared with the results of the *rec*A-*pyr*H metabarcoding analysis conducted on the same 16 plated clam samples (Figure 6). In this analysis, 16S RNA gene results were not included because they only perform taxonomic identification of the vibrios bacterial community up to the genus level [52].

The shotgun metagenomics data more accurately characterized microbial community composition. Specifically, shotgun metagenomics offered a more complete picture of the *Vibrio* and non-*Vibrio* biodiversity (Figure 6, Figure S5, Supplementary Materials) present in Manila clam microbiota. Similarly to the results achieved, other studies demonstrated that shotgun metagenomics provides a better resolution on the species taxonomic level compared to metabarcoding [53,54]. Consequently, as expected, *rec*A*-pyr*H metabarcoding identified a limited number of *Vibrio* species in clam samples plated on MA and TCBS media and on homogenate samples (Figures S3 and S4, Supplementary Materials, Figure 6). This finding suggested that the limit of metabarcoding in *Vibrio* spp. detection is not only related to the culture-dependent and -independent methods used to process clam samples; in fact, a similar number of Vibrionaceae and *Vibrio* species were identified in homogenates and on clam samples plated on MA and TCBS media (Figure 6). This result confirmed the bias of the PCR-based approach in the amplification of different species and the difficulties in designing completely inclusive genus-specific universal primers [17,18,19].

The main human pathogenic *Vibrio* species, such as *V. alginolyticus*, *V. cholerae*, *V. parahaemolytic*us and *V. vulnificus*, identified by shotgun metagenomics were not found in all of the samples with *rec*A*-pyr*H metabarcoding. This result confirms the higher level of sensitivity of shotgun metagenomics with respect to metabarcoding for the detection of these species, which are of particular concern for human health, that frequently occur in low concentrations. In addition, it is important to point out that the application of metagenomics on plated clam samples offered the advantage of detecting the main viable human pathogenic *Vibrio* species, such as *V. alginolyticus*, *V. cholerae*, *V. parahaemolytic*us and *V. vulnificus*, in an alive and, consequently, potentially virulent state.

Nevertheless, metabarcoding identified *P. gaetbulicola*/*rosenbergii*, *V. cidicii*, *V. fluvialis*, *V. ponticus*, *V.sp.Scap.24* and *V.sp.THAF100*, which were not detected by shotgun metagenomics (Figure 6). Of these, *P. gaetbulicola*/*rosenbergii*, *V.sp.Scap.24* and *V. ponticus* were detected only in clam samples plated on TCBS medium (Figure 5). It is likely that these six species belong to that part of rare bacterial biodiversity for which shotgun metagenomics presents a limited resolution. In accordance with these results, Srivathsan et al. [55] achieved better detection of rare taxa by using metabarcoding with respect to shotgun metagenomics.

Shotgun metagenomics is currently applied to map microbial contamination in the food industry [24,53,56]. This study showed the usefulness of carrying out a microbial community characterization using culture-dependent shotgun metagenomics. Specifically, the application of shotgun metagenomics on plated clam samples overcomes the problem of host DNA that could overwhelm microbiota DNA. Moreover, this approach offered a less time-consuming characterization of the alive fraction of bacteria down to the species taxonomic level. For this reason, the cultural-dependent shotgun metagenomics reduced the cost of the analysis to one required by the metabarcoding approach. Given this, culture-dependent shotgun metagenomics could be applied in the seafood industry, for example, screening techniques to prevent human vibriosis that could be spread by contaminated seafood products such as fish, crustaceans or shellfish. In addition, in the aquaculture sector, both metabarcoding and shotgun metagenomics could also be useful for the characterization of the biofilm microbial composition, which could comprise *Vibrio* species pathogenic for humans and species pathogenic to farmed animals [57].

## 5. Conclusions

In the current study, the culture-dependent method coupled with the culture-independent method was a valid tool to achieve the characterization of several *Vibrio* species in the *Ruditapes philippinarum* microbiota. The culture-dependent and -independent methods showed a comparable reliability for the detection of *V. parahaemolyticus* and *V. vulnificus*. On the contrary, the two methods proved to be complementary for the detection of *V. alginolyticus* and *V. cholerae*. The obtained results suggested a reassessment of MA as a suitable medium for the recovery of Vibrios, including the main human *Vibrio* pathogenic species. Moreover, the study showed that metabarcoding, applied on the homogenate samples and on clam samples plated on MA media, could represent a useful screening approach for the prevention of human vibriosis related to contaminated seafood. Furthermore, the comparison of the shotgun metagenomics and metabarcoding results highlighted the higher number of species detected by shotgun metagenomics during the *Vibrio* species detection. In particular, the application of cultural-dependent shotgun metagenomics offered a reliable and accurate characterization of the alive fraction of bacteria down to the species taxonomic level.

## Supplementary Materials

The following are available online at https://www.mdpi.com/article/10.3390/foods10061271/s1, Table S1: Summary of the total 54 clam samples collected during summer and winter seasons. Samples are subdivided according to the culture-independent and -dependent methods. Clam samples were plated on the following: A: MA22 °C, Marine agar incubated at 22 °C; B: TCBS 22 °C, Thiosulfate-citrate-bile salts-sucrose agar incubated at 22 °C; C: CV 22 °C, CHROMagar *Vibrio* media incubated at 22 °C: D: TCBS 37 °C, Thiosulfate-citrate-bile salts-sucrose agar incubated at 37 °C. Sampling sites: CH: Chioggia; CO: Colmata; GO: Goro; MA: Marano; PM: Porto Marghera; SC: Scardovari. 1: Pre-depuration samples; 2: Post-depuration samples. Table S2: Fleiss’ kappa agreement of *Vibrio* species detection in homogenate and clam samples plated and incubated at 22 °C on MA and TCBS media according to *rec*A-*pyr*H metabarcoding. The *Vibrio* species with a significant statistical Fleiss’ kappa are shown in bold. Fleiss’ kappa values agreement interpretation: k < 0—poor; k = 0.01–0.20—slight; k = 0.21–0.40—fair; k = 0.41–0.60—moderate; k = 0.61–0.80—substantial; k = 0.81–1—almost perfect; Table S3. Resulting sequencing depth obtained by the shotgun metagenomics; Figure S1: Rarefaction curve of homogenate and plated clam samples reads obtained by 16S sequencing. OM: Homogenate clam samples; MA 22 °C: clam samples plated on marine agar and incubated at 22 °C; TCBS 22 °C: clam samples plated on Thiosulfate-citrate-bile salts-sucrose agar and incubated at 22 °C; CV 22 °C: clam samples plated on CHROMagar *Vibrio* media and incubated at 22 °C. Figure S2: *rec*A-*pyr*H amplicon-based microbial principal coordinate analysis (PCoA) representation of homogenate and plated clam samples according to Jaccard index. In PCoA representation, the season factor corresponding to the collection time of clam samples is also reported. OM: Homogenate clam samples; MA 22 °C: clam samples plated on marine agar and incubated at 22 °C; TCBS 22 °C: clam samples plated on thiosulfate-citrate-bile salts-sucrose agar and incubated at 22 °C; CV 22 °C: clam samples plated on CHROMagar *Vibrio* media and incubated at 22 °C. Figure S3: Venn diagram showing the number of species identified in clam samples plated in MA medium and incubated at 22 °C, according to shotgun metagenomics and *rec*A-*pyr*H metabarcoding. The table reports the details of the 16 species shared between the two methods. MA 22 °C: clam samples plated on marine agar media and incubated at 22 °C. Figure S4: Venn diagram showing the number of species identified in clam samples plated in TCBS medium and incubated at 22° C, according to shotgun metagenomics and *rec*A-*pyr*H metabarcoding. TCBS 22 °C: clam samples plated on thiosulfate-citrate-bile salts-sucrose agar and incubated at 22 °C. Figure S5: Heat map showing the presence (green) and absence (red) of marine bacteria, *Vibrio* species excluded, found in clam samples according to metagenomics and *rec*A-*py*rH metabarcoding.

## References

[1] Romalde J.L., Diéguez A.L., Lasa A., Balboa S. (2014). New Vibrio species associated to molluscan microbiota: A review. Front. Microbiol..

[2] West P.A. (1989). The human pathogenic vibrios—A public health update with environmental perspectives. Epidemiol. Infect..

[3] Jiang Y., Chu Y., Xie G., Li F., Wang L., Huang J., Zhai Y., Yao L. (2019). Antimicrobial resistance, virulence and genetic relationship of Vibrio parahaemolyticus in seafood from coasts of Bohai Sea and Yellow Sea, China. Int. J. Food Microbiol..

[4] Passalacqua P.L., Zavatta E., Bignami G., Serraino A., Serratore P., Studiorum- A.M., Emilia O. (2016). Occurrence of Vibrio parahaemolyticus, Vibrio cholerae and Vibrio vulnificus in the clam Ruditapes philippinarum (Adams & Reeve, 1850) from Emilia Romagna and Sardinia, Italy. Ital. J. Food Saf..

[5] Baker-Austin C., Trinanes J., Gonzalez-Escalona N., Martinez-Urtaza J. (2017). Non-Cholera Vibrios: The Microbial Barometer of Climate Change. Trends Microbiol..

[6] Bonnin-Jusserand M., Copin S., Le Bris C., Brauge T., Gay M., Brisabois A., Grard T., Midelet-Bourdin G. (2019). Vibrio species involved in seafood-borne outbreaks (Vibrio cholerae, *V. parahaemolyticus* and *V. vulnificus*): Review of microbiological versus recent molecular detection methods in seafood products. Crit. Rev. Food Sci. Nutr..

[7] Colwell R.R. (2005). Oceans and Health: Pathogens in the Marine Environment.

[8] Kriem M.R., Banni B., El Bouchtaoui H., Hamama A., El Marrakchi A., Chaouqy N., Robert-Pillot A., Quilici M.L. (2015). Prevalence of Vibrio spp. in raw shrimps (Parapenaeus longirostris) and performance of a chromogenic medium for the isolation of Vibrio strains. Lett. Appl. Microbiol..

[9] Carraro L., Maifreni M., Bartolomeoli I., Martino M.E., Novelli E., Frigo F., Marino M., Cardazzo B. (2011). Comparison of culture-dependent and -independent methods for bacterial community monitoring during Montasio cheese manufacturing. Res. Microbiol..

[10] Nadkarni M.A., Martin F.E., Hunter N., Jacques N.A. (2009). Methods for optimizing DNA extraction before quantifying oral bacterial numbers by real-time PCR. FEMS Microbiol. Lett..

[11] Malara D., Mielke C., Oelgemöller M., Senge M.O., Heimann K. (2017). Sustainable water treatment in aquaculture—Photolysis and photodynamic therapy for the inactivation of Vibrio species. Aquac. Res..

[12] Liu Y., Zhong Q., Wang J., Lei S. (2018). Enumeration of Vibrio parahaemolyticus in VBNC state by PMA-combined real-time quantitative PCR coupled with confirmation of respiratory activity. Food Control.

[13] Orruño M., Kaberdin V.R., Arana I. (2017). Survival strategies of Escherichia coli and Vibrio spp.: Contribution of the viable but nonculturable phenotype to their stress-resistance and persistence in adverse environments. World J. Microbiol. Biotechnol..

[14] Lee J., Azizah R.N., Kim K. (2020). Comparative evaluation of three agar media-based methods for presumptive identi fi cation of seafood-originated Vibrio parahaemolyticus strains. Food Control.

[15] Fenske G.J., Ghimire S., Antony L., Christopher-Hennings J., Scaria J. (2020). Integration of culture-dependent and independent methods provides a more coherent picture of the pig gut microbiome. FEMS Microbiol. Ecol..

[16] Kirchberger P.C., Orata F.D., Nasreen T., Kauffman K.M., Tarr C.L., Case R.J., Polz M.F., Boucher Y.F. (2020). Culture-independent tracking of Vibrio cholerae lineages reveals complex spatiotemporal dynamics in a natural population. Environ. Microbiol..

[17] Jesser K.J., Rachel T. (2018). Noble Vibrio Ecology in the Neuse River Estuary, North Carolina, Characterized by Next-Generation Amplicon Sequencing of the Gene Encoding Heat Shock Protein 60 (hsp60). Appl. Environ. Microbiol..

[18] King W.L., Jenkins C., Go J., Siboni N., Seymour J.R., Labbate M. (2019). Characterisation of the Pacific Oyster Microbiome During a Summer Mortality Event. Microb. Ecol..

[19] Zampieri A., Carraro L., Cardazzo B., Milan M., Babbucci M., Smits M., Boffo L., Fasolato L. (2020). Depuration processes affect the Vibrio community in the microbiota of the Manila clam, Ruditapes philippinarum. Environ. Microbiol..

[20] Escobar-Zepeda A., Sanchez-Flores A., Quirasco Baruch M. (2016). Metagenomic analysis of a Mexican ripened cheese reveals a unique complex microbiota. Food Microbiol..

[21] Soejima T., Iida K.I., Qin T., Taniai H., Seki M., Yoshida S.I. (2008). Method to detect only live bacteria during PCR amplification. J. Clin. Microbiol..

[22] Sidstedt M., Rådström P., Hedman J. (2020). PCR inhibition in qPCR, dPCR and MPS—Mechanisms and solutions. Anal. Bioanal. Chem..

[23] Pereira-Marques J., Hout A., Ferreira R.M., Weber M., Pinto-Ribeiro I., Van Doorn L.J., Knetsch C.W., Figueiredo C. (2019). Impact of host DNA and sequencing depth on the taxonomic resolution of whole metagenome sequencing for microbiome analysis. Front. Microbiol..

[24] McHugh A., Feehily C., Fenelon M., Gleeson D., Hill C., Cotter P. (2020). Tracking the Dairy Microbiota from Farm Bulk Tank. Am. Soc. Microbiol..

[25] Rubiola S., Chiesa F., Dalmasso A., Di Ciccio P., Civera T. (2020). Detection of Antimicrobial Resistance Genes in the Milk Production Environment: Impact of Host DNA and Sequencing Depth. Front. Microbiol..

[26] Anguita-Maeso M., Olivares-García C., Haro C., Imperial J., Navas-Cortés J.A., Landa B.B. (2020). Culture-Dependent and Culture-Independent Characterization of the Olive Xylem Microbiota: Effect of Sap Extraction Methods. Front. Plant Sci..

[27] Ndoye B., Rasolofo E.A., LaPointe G., Roy D. (2011). A review of the molecular approaches to investigate the diversity and activity of cheese microbiota. Dairy Sci. Technol..

[28] Udyavar V., Muthappa D.M., Venugopal M., Animal K.V. (2016). Prevalence and Genomic Characterization of Vibrio parahaemolyticus isolated from Molluscan Shellfish and their Inhabiting Water of Coastal Karnataka, India. Int. J. Curr. Microbiol. Appl. Sci..

[29] Milan M., Carraro L., Fariselli P., Martino M.E., Cavalieri D., Vitali F., Bo L., Patarnello T., Bargelloni L., Cardazzo B. (2018). Microbiota and environmental stress: How pollution a ff ects microbial communities in Manila clams. Aquat. Toxicol..

[30] Wood D.E., Lu J., Langmead B. (2019). Improved metagenomic analysis with Kraken 2. Genome Biol..

[31] Lu J., Breitwieser F.P., Thielen P., Salzberg S.L. (2017). Bracken: Estimating species abundance in metagenomics data. PeerJ.

[32] Zakrzewski M., Proietti C., Ellis J.J., Hasan S., Brion M.J., Berger B., Krause L. (2017). Calypso: A user-friendly web-server for mining and visualizing microbiome-environment interactions. Bioinformatics.

[33] Feder I., Nietfeld J.C., Galland J., Yeary T., Sargeant J.M., Oberst R., Tamplin M.L., Luchansky J.B. (2001). Comparison of cultivation and PCR-hybridization for detection of Salmonella in porcine fecal and water samples. J. Clin. Microbiol..

[34] Uritskiy G.V., DiRuggiero J., Taylor J. (2018). MetaWRAP—A flexible pipeline for genome-resolved metagenomic data analysis. Microbiome.

[35] Senderovich Y., Izhaki I., Halpern M. (2010). Fish as reservoirs and vectors of Vibrio cholerae. PLoS ONE.

[36] Serratore P., Ostanello F., Serraino A., Giacometti F. (2016). First multi-year retrospective study on Vibrio parahaemolyticus and Vibrio vulnificus prevalence in Ruditapes philippinarum in Sacca di Goro, Italy. Ital. J. Food Saf..

[37] Xu Y.G., Sun L.M., Wang Y.S., Chen P.P., Liu Z.M., Li Y.J., Tang L.J. (2017). Simultaneous detection of Vibrio cholerae, Vibrio alginolyticus, Vibrio parahaemolyticus and Vibrio vulnificus in seafood using dual priming oligonucleotide (DPO) system-based multiplex PCR assay. Food Control.

[38] Zapka C., Leff J., Henley J., Tittl J., De Nardo E., Butler M., Griggs R., Fierer N., Edmonds-Wilson S. (2017). Comparison of standard culture-based method to culture-independent method for evaluation of hygiene effects on the hand microbiome. MBio.

[39] Di Pinto A., Terio V., Novello L., Tantillo G. (2011). Comparison between thiosulphate-citrate-bile salt sucrose (TCBS) agar and CHROMagar Vibrio for isolating Vibrio parahaemolyticus. Food Control.

[40] Zhang X.H., Ahmad W., Yu X., Jixiang Z., Brian C. (2020). Viable but nonculturable bacteria and their resuscitation: Implications for cultivating uncultured marine microorganisms. Mar. Life Sci. Technol..

[41] Kakizaki E., Takahama K., Seo Y., Kozawa S., Sakai M., Yukawa N. (2008). Marine bacteria comprise a possible indicator of drowning in seawater. Forensic Sci. Int..

[42] Nigro O.D., Steward G.F. (2015). Differential specificity of selective culture media for enumeration of pathogenic vibrios: Advantages and limitations of multi-plating methods. J. Microbiol. Methods.

[43] Kaevska M., Videnska P., Sedlar K., Slana I. (2016). Seasonal changes in microbial community composition in river water studied using 454-pyrosequencing. Springerplus.

[44] Wang Y., Liu Y., Wang J., Luo T., Zhang R., Sun J., Zheng Q., Jiao N. (2019). Seasonal dynamics of bacterial communities in the surface seawater around subtropical Xiamen Island, China, as determined by 16S rRNA gene profiling. Mar. Pollut. Bull..

[45] Tagliavia M., Salamone M., Bennici C., Quatrini P., Cuttitta A. (2019). A modified culture medium for improved isolation of marine vibrios. Microbiologyopen.

[46] Pezzlo M., Valter P.J., Burns M.J. (1979). Wound infection associated with Vibrio alginolyticus. Am. J. Clin. Pathol..

[47] Li R., Lu J., Duan H., Yang J., Tang C. (2020). Biofilm inhibition and mode of action of epigallocatechin gallate against Vibrio mimicus. Food Control.

[48] Elhadi N., Radu S., Chen C.H., Nishibuchi M. (2004). Prevalence of potentially pathogenic vibrio species in the seafood marketed in Malaysia. J. Food Prot..

[49] Zarei M., Borujeni M.P., Jamnejad A., Khezrzadeh M. (2012). Seasonal prevalence of Vibrio species in retail shrimps with an emphasis on Vibrio parahaemolyticus. Food Control.

[50] Beaz-Hidalgo R., Balboa S., Romalde J.L., Figueras M.J. (2010). Diversity and pathogenecity of Vibrio species in cultured bivalve molluscs. Environ. Microbiol. Rep..

[51] Sharpton T.J. (2014). An introduction to the analysis of shotgun metagenomic data. Front. Plant Sci..

[52] Thompson F.L., Gevers D., Thompson C.C., Dawyndt P., Naser S., Hoste B., Munn C.B., Swings J. (2005). Phylogeny and Molecular Identification of Vibrios on the Basis of Multilocus Sequence Analysis. Appl. Environ. Microbiol..

[53] Grützke J., Malorny B., Hammerl J.A., Busch A., Tausch S.H., Tomaso H., Deneke C. (2019). Fishing in the soup—Pathogen detection in food safety using metabarcoding and metagenomic sequencing. Front. Microbiol..

[54] Srivathsan A., Ang A., Vogler A.P., Meier R. (2016). Fecal metagenomics for the simultaneous assessment of diet, parasites, and population genetics of an understudied primate. Front. Zool..

[55] Srivathsan A., Sha J.C.M., Vogler A.P., Meier R. (2015). Comparing the effectiveness of metagenomics and metabarcoding for diet analysis of a leaf-feeding monkey (Pygathrix nemaeus). Mol. Ecol. Resour..

[56] De Filippis F., Valentino V., Alvarez-Ordóñez A., Cotter P.D., Ercolini D. (2021). Environmental microbiome mapping as a strategy to improve quality and safety in the food industry. Curr. Opin. Food Sci..

[57] Arunkumar M., LewisOscar F., Thajuddin N., Pugazhendhi A., Nithya C. (2020). In vitro and in vivo biofilm forming Vibrio spp: A significant threat in aquaculture. Process Biochem..

