# Supplementary material for: Combining Culture-Dependent and Culture-Independent Methods: New Methodology Insight on the Vibrio Community of Ruditapes philippinarum
Source: Foods. 2021 Jun 3;10(6):1271. doi: 10.3390/foods10061271 (PMC8228196; doi:10.3390/foods10061271)
Supplement: Supplementary file 1 [file foods-10-01271-s001.zip › foods-1237487-supplementary.pdf]

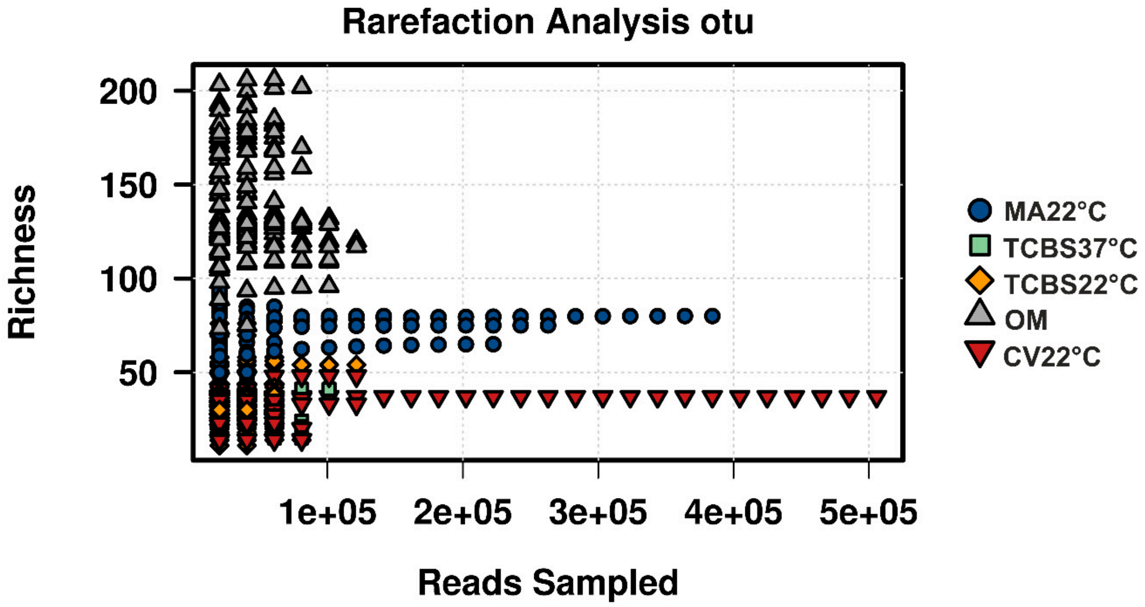

Figure S1. Rarefaction curve of homogenate and plated clam samples reads obtained by 16S sequencing. OM: Homogenate clam samples; MA 22 °C: clam samples plated on marine agar and incubated at 22 °C; TCBS 22 °C: clam samples plated on Thiosulfate-citrate-bile salts-sucrose agar and incubated at 22 °C; CV 22 °C: clam samples plated on CHROMagar Vibrio media and incubated at 22 °C.

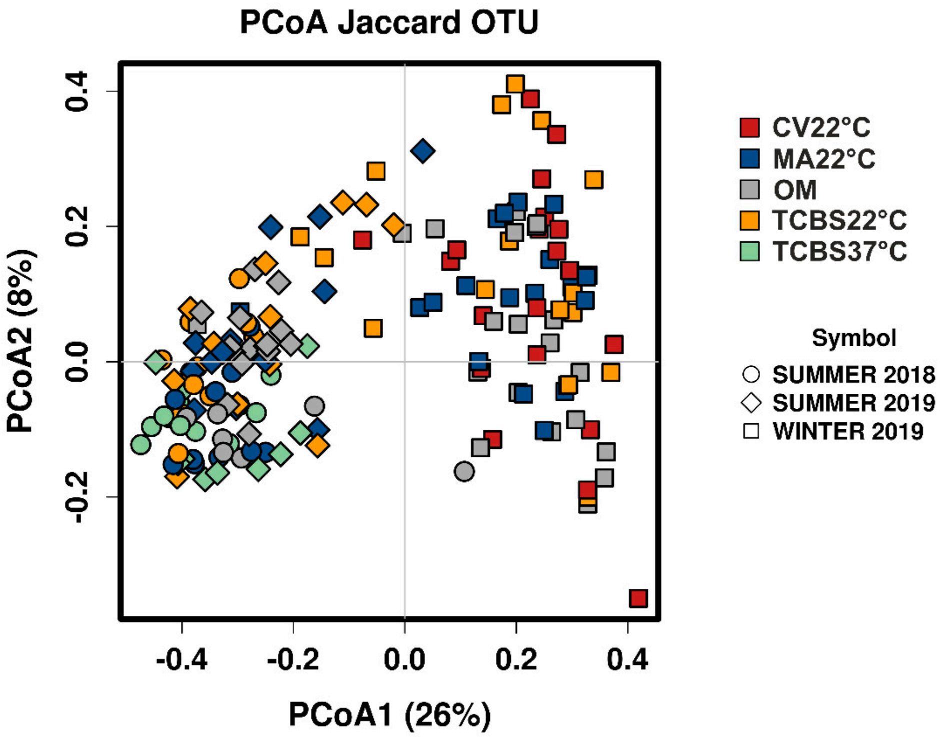

Figure S2. *rec*A-*pyr*H amplicon-based microbial principal coordinate analysis (PCoA) representation of homogenate and plated clam samples according to Jaccard index. In PCoA representation, the season factor corresponding to the collection time of clam samples is also reported. OM: Homogenate clam samples; MA 22 °C: clam samples plated on marine agar and incubated at 22 °C; TCBS 22 °C: clam samples plated on thiosulfate-citrate-bile salts-sucrose agar and incubated at 22 °C; CV 22 °C: clam samples plated on CHROMagar Vibrio media and incubated at 22 °C.

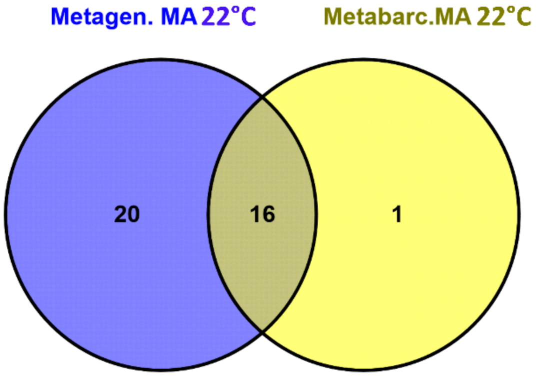

16 shared species
Aliivibrio wodanis
Photobacterium damselae
Photobacterium profundum
Vibrio astriarenae
Vibrio campbellii
Vibrio crassostreae
Vibrio cyclitrophicus
Vibrio diabolicus
Vibrio furnissii
Vibrio harveyi
Vibrio mediterranei
Vibrio parahaemolyticus
Vibrio rotiferianus
Vibrio splendidus
Vibrio tapetis
Vibrio vulnificus

Figure S3. Venn diagram showing the number of species identified in clam samples plated in MA medium and incubated at 22 °C, according to shotgun metagenomics and *rec*A-*pyr*H metabarcoding. The table reports the details of the 16 species shared between the two methods. MA 22 °C: clam samples plated on marine agar media and incubated at 22 °C.

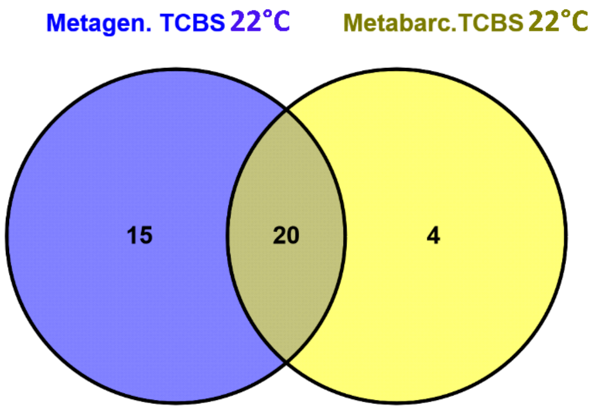

20 shared species
Photobacterium damselae
Vibrio alfacsensis
Vibrio anguillarum
Vibrio astriarenae
Vibrio breoganii
Vibrio campbellii
Vibrio chagasii
Vibrio cyclitrophicus
Vibrio diabolicus
Vibrio furnissii
Vibrio harveyi
Vibrio jasicida
Vibrio mediterranei
Vibrio owensii
Vibrio parahaemolyticus
Vibrio rotiferianus
Vibrio sp. THAF190c
Vibrio splendidus
Vibrio tapetis
Vibrio vulnificus

Figure S4. Venn diagram showing the number of species identified in clam samples plated in TCBS medium and incubated at 22° C, according to shotgun metagenomics and *rec*A-*pyr*H metabarcoding. TCBS 22 °C: clam samples plated on thiosulfate-citrate-bile salts-sucrose agar and incubated at 22 °C.

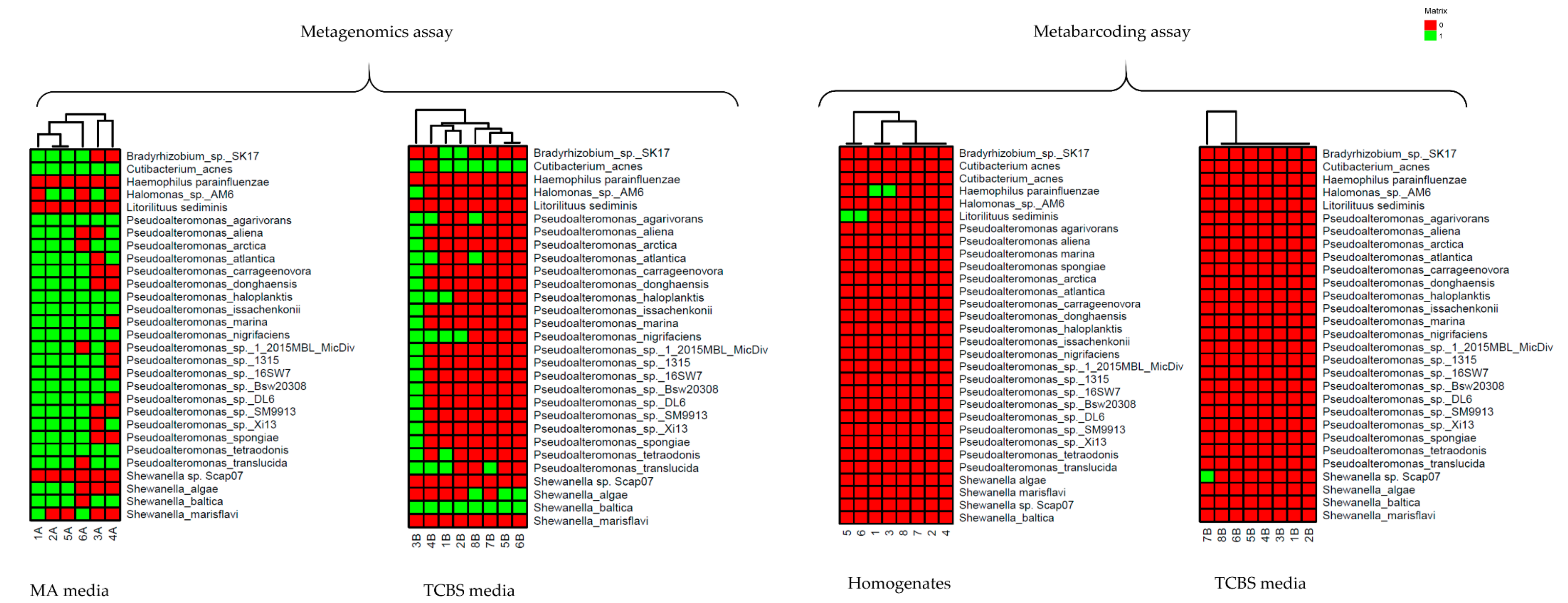

Figure S5. Heat map showing the presence (green) and absence (red) of marine bacteria, *Vibrio* species ex-cluded, found in clam samples according to metagenomics and *rec*A-*pyr*H metabarcod-ing.

Table S1. Summary of the total 54 clam samples collected during summer and winter seasons. Samples are subdivided according to the culture-independent and -dependent methods. Clam samples were plated on the following: A: MA22 °C, Marine agar incubated at 22 °C; B: TCBS 22 °C, Thiosulfate-citrate-bile salts-sucrose agar incubated at 22 °C; C: CV 22 °C, CHROMagar Vibrio media incubated at 22 °C: D: TCBS 37 °C, Thiosulfate-citrate-bile salts-sucrose agar incubated at 37 °C. Sampling season: Summer; Winter. Sampling sites: CH: Chioggia; CO: Colmata; GO: Goro; MA: Marano; PM: Porto Marghera; SC: Scardovari. 1: Pre-depuration samples; 2: Post-depuration samples.

			**CULTURE-INDEPENDENT**	**CULTURE-DEPENDENT**					
				**MA22 °C**		**TCBS 22 °C**		**TCBS 37°C**	**CV22 °C**
**Sites**	**Season**	**Depuration**	**Homogenates**	**Metagen.**	**Metabarc.**	**Metagen.**	**Metabarc.**	**Metabarc.**	**Metabarc.**
CH	Summer	1	**1**	1A	1A	1B	1B	1D	
CH	Summer	2	**2**	2A	2A	2B	2B	2D	
CH	Winter	1	**3**	*	3A	3B	3B		3C
CH	Winter	2	**4**	4A	4A	4B	4B		4C
PM	Summer	1	**5**	5A	5A	5B	5B	5D	
PM	Summer	2	**6**	6A	6A	6B	6B	6D	
PM	Winter	1	**7**	*	7A	7B	7B		7C
PM	Winter	2	**8**	*	8A	8B	8B		8C
SC	Summer	1			9A		9B	9D	
SC	Summer	2			10A		10B	10D	
CO	Summer	1	**11**		11A		11B	11D	
CO	Summer	2	**12**		12A		12B	12D	
GO	Summer	1			13A		13B	13D	
GO	Summer	2			14A		14B	14D	
MA	Summer	1	**15**		15A		15B	15D	
MA	Summer	2	**16**		16A		16B	16D	
CH	Winter	1	**17**		17A		17B		17C
CH	Winter	2	**18**		18A		18B		18C
CH	Winter	1	**19**		19A		19B		19C
CH	Winter	2	**20**		20A		20B		20C
CH	Winter	1	**21**		21A		21B		21C
CH	Winter	2	**22**		22A		22B		22C

PM	Winter	1	**23**	23A	23B		23C
PM	Winter	2	**24**	24A	24B		24C
SC	Winter	1	**25**	25A	25B		25C
SC	Winter	2	**26**	26A	26B		26C
SC	Winter	1	**27**	27A	27B		27C
SC	Winter	2	**28**	28A	28B		28C
CO	Winter	1	**29**	29A	29B		29C
CO	Winter	2	**30**	30A	30B		30C
CO	Winter	1	**31**	31A	31B		31C
CO	Winter	2	**32**	32A	32B		32C
GO	Winter	1	**33**	33A	33B		33C
GO	Winter	2	**34**	34A	34B		34C
GO	Winter	1	**35**	35A	35B		35C
GO	Winter	2	**36**	36A	36B		36C
MA	Winter	1	**37**	37A	37B		37C
MA	Winter	2	**38**	38A	38B		38C
MA	Winter	1	**39**	39A	39B		39C
MA	Winter	2	**40**	40A	40B		40C
CH	Summer	1	**41**	41A	41B	41D	
CH	Summer	2	**42**	42A	42B	/	
CH	Summer	1	**43**	43A	43B	43D	
CH	Summer	2	**44**	44A	44B	44D	
PM	Summer	1	**45**	45A	45B	45D	
PM	Summer	2	**46**	46A	46B	46D	
SC	Summer	1	**47**	47A	/	/	
SC	Summer	2	**48**	48A	48B	48D	
CO	Summer	1	**49**	49A	49B	49D	
CO	Summer	2	**50**	50A	50B	50D	
GO	Summer	1	**51**	51A	/	/	
GO	Summer	2	**52**	52A	52B	52D	
MA	Summer	1	**53**	53A	53B	53D	

MA	Summer	2	**54**	54A	54B	/

* Metagenomics samples not included in the study because of the low quality reads

/ Plated clam samples not included on the analysis because the number of grown colonies was below 10

Metagen.: Metgenomics

Metabarc.: Metabarcoding

Table S2. Fleiss' kappa agreement of *Vibrio* species detection in homogenate and clam samples plated and incubated at 22 °C on MA and TCBS media according to *rec*A-*pyr*H metabarcoding.The *Vibrio* species with a significant statistical Fleiss' kappa are shown in bold. Fleiss ' kappa values agreement interpretation: k < 0—poor; k = 0.01–0.20—slight; k = 0.21–0.40—fair; k = 0.41–0.60—moderate; k = 0.61–0.80—substantial; k = 0.81–1—almost perfect

Species	Fleiss 'kappa	Agreement	*p*-value
Aliivibrio fischeri	0.051	Slight	0.544
Aliivibrio wodanis	0.133	Slight	0.111
Photobacterium damselae	**0.226**	**Fair**	**0.007**
Photobacterium gaetbulicola/ Photobacterium rosenbergii	**0.409**	**Moderate**	**0.001**
Photobacterium profundum	**0.493**	**Moderate**	**0.001**
Vibrio alfacsensis	**0.4**	**Fair**	**0.001**
Vibrio alginolyticus	0.052	Slight	0.534
Vibrio anguillarum	0.074	Slight	0.378
Vibrio astriarenae	**0.57**	**Moderate**	**0.001**
Vibrio atlanticus	0.114	Slight	0.171
Vibrio azureus	**0.493**	**Moderate**	**0.001**
Vibrio breoganii	**0.181**	**Slight**	**0.029**
Vibrio campbellii	0.14	Slight	0.09
Vibrio casei	-0.07	Poor	0.933
Vibrio chagasii	0.15	Slight	0.072
Vibrio cholerae	0.031	Slight	0.73
Vibrio cidicii	-0.07	Poor	0.933
Vibrio crassostreae	0.108	Slight	0.195
Vibrio cyclitrophicus	-0.055	Poor	0.519
Vibrio diabolicus	**0.367**	**Fair**	**0.001**
Vibrio europaeus	-0.014	Poor	0.843
Vibrio fluvialis	-0.042	Poor	0.647
Vibrio furnissii	0.107	Slight	0.199
Vibrio harveyi	**0.718**	**Substantial**	**0.001**
Vibrio jasicida	-0.036	Poor	0.666
Vibrio mediterranei	**0.676**	**Substantial**	**0.001**
Vibrio nigripulchritudo	-0.014	Poor	0.866
Vibrio owensii	0.033	Slight	0.696
Vibrio panuliri	0.133	Slight	0.199
Vibrio parahaemolyticus	**0.477**	**Moderate**	**0.001**
Vibrio ponticus	-0.021	Poor	0.966
Vibrio rotiferianus	**0.543**	**Moderate**	**0.001**
Vibrio rumoiensis	-0.021	Poor	0.966
Vibrio scophthalmi	-0.026	Poor	0.866
Vibrio sp. Scap24	-0.021	Poor	0.966
Vibrio sp. THAF100	-0.014	Poor	0.966
Vibrio sp. THAF190c	0.107	Slight	0.299
Vibrio splendidus	**0.379**	**Fair**	**0.001**
Vibrio tapetis	**0.358**	**Fair**	**0.001**
Vibrio tubiashii	**0.493**	**Moderate**	**0.001**

Vibrio vulnificus	**0.454**	**Moderate**	**0.001**

Table S3. Resulting sequencing depth obtained by the shotgun metagenomics

Sample	Total raw reads
1A	14067580
1B	10467200
2A	7223358
2B	12635596
3B	11110382
4A	11772854
4B	9922952
5A	9991516
5B	4175460
6A	504008
6B	8678986
7B	11407522
8B	9820130
